# Supplementary material for: Parallel pathways for sound processing and functional connectivity among layer 5 and 6 auditory corticofugal neurons
Source: eLife. 2019 Feb 8;8:e42974. doi: 10.7554/eLife.42974 (PMC6384027; doi:10.7554/eLife.42974)
Supplement: Supplementary file 1. — Supplemental Table S1. Summary table detailing sample sizes and parameter values for all sensory characterization comparisons (Figures 3–5). Supplemental Table S2. Summary table detailing sample sizes for all connectivity comparisons (Figures 6–7). Supplemental Table S3. Summary table detailing firing rates evoked by all presented stimuli. [file elife-42974-supp1.docx]

**Supplemental Table S1.** Summary table detailing sample sizes and parameter values for all sensory characterization comparisons (Figures 3-5).

| **Comparison** | **Number of Units** | | | | **Median Value ± 95% CI** | | | |
| --- | --- | --- | --- | --- | --- | --- | --- | --- |
|  | **L5** | **L6** | **L5 CF** | **L6 CT** | **L5** | **L6** | **L5 CF** | **L6 CT** |
| Pure tone stimuli: FRA bandwidth (Figure 3C) | 174/509 | 172/625 | 44/132 | 27/83 | 1 ± 0.07 | 1 ± 0.07 | 1.1 ± 0.13 | 0.9 ± 0.17 |
| Pure tone stimuli: latency (Figure 3D) |  |  |  |  | 20 ± 0.83 | 13 ± 1.08 | 21 ± 1.79 | 13 ± 1.14 |
| Random stimuli: sparsity (Figure 3G) | 211/509 | 274/625 | 50/132 | 38/83 | 0.39 ± 0.04 | 0.46 ± 0.03 | 0.28 ± 0.06 | 0.41 ± 0.1 |
| DRC stimuli: STRF predictive power (Figure 5B) | 372/509 | 295/625 | 81/132 | 51/83 | 0.06 ± 0.01 | 0.1 ± 0.02 | 0.06 ± 0.03 | 0.09 ± 0.05 |
| DRC stimuli: CM predictive power (Figure 5B) |  |  |  |  | 0.08 ± 0.01 | 0.12 ± 0.02 | 0.08 ± 0.02 | 0.12 ± 0.05 |

**Supplemental Table S2.** Summary table detailing sample sizes for all connectivity comparisons (Figures 6-7).

| **Comparison** | **Number of Units** | | | |
| --- | --- | --- | --- | --- |
|  | **L5 CF -> RS** | **L5 CF -> FS** | **L6 CT -> RS** | **L6 CT -> FS** |
| Normalized firing rate (Figure 6D) | 501 | 96 | 296 | 63 |
|  | **Number of Pairs** | | | |
|  | **L5 CF <-> RS** | **L5 CF <-> FS** | **L6 CT <-> RS** | **L6 CT <-> FS** |
| CCov integral (Figure 7D) | 2353 | 448 | 981 | 209 |
| Asymmetry index (Figure 7H) | 189 | 56 | 128 | 54 |

**Supplemental Table S3.** Summary table detailing firing rates evoked by all presented stimuli.

| **Stimulus** | **Evoked Firing Rate (Hz)** | | | |
| --- | --- | --- | --- | --- |
|  | **L5** | **L6** | **L5 CF** | **L6 CT** |
| Pure tones | 19.32 ± 2.41 | 20.15 ± 2.91 | 22.05 ± 4.8 | 20.38 ± 8.33 |
| Random stimuli | 21.75 ± 2.65 | 23 ± 2.79 | 19.25 ± 4.58 | 23.75 ± 6.66 |
| DRC | 7.6 ± 0.69 | 7.15 ± 0.68 | 9.05 ± 1.51 | 10.2 ± 2.49 |
